# An exploration of evidence-based practice work files for occupational therapy students during clinical placements: a descriptive cross-sectional study

**DOI:** 10.1186/s12909-020-02178-2

**Published:** 2020-08-06

**Authors:** Susanne Grødem Johnson, Else Britt Bruset, Kari Margrete Hjelle, Malin Mongs, Unni Sveen

**Affiliations:** 1 Department of Health and Function, Faculty of Health and Social Sciences, Western Norway University of Applied Sciences, Inndalsveien 28, 5063 Bergen, Norway; 2 Department of Occupational Therapy, Oslo Metropolitan University, PO Box 4, St. Olavs plass, NO-0130 Oslo, Norway; 3 Department of Occupational Therapy, Oslo University Hospital, Rikshospitalet, Postboks 4950 Nydalen, 0424 Oslo, Norway

**Keywords:** EBP work file, EBP beliefs scale, EBP implementation scale, Occupational therapy education, Clinical placement

## Abstract

**Background:**

Bachelor students of occupational therapy are expected to work in accordance with evidence-based practice (EBP). The EBP work file, a learning tool in a Word document format, covering all steps in the EBP process, is an approach to teaching and learning EBP. The aim of this study was to examine the attitudes and behaviours of occupational therapy students’ in relation to applying evidence-based practice during their second-year clinical placement. We compared cohorts who received training in EBP work files with those who did not receive such training.

**Methods:**

A descriptive, cross-sectional comparative study was conducted. Five cohorts of second-year occupational therapy students took part in the study. The students answered two questionnaires, the EBP Beliefs Scale and the EBP Implementation Scale, after completing their second-year clinical placement. The analysis was based on descriptive statistics and calculation of the frequencies, percentages, mean and standard deviations of all participating students’ scores across both questionnaires. ANOVA with Bonferroni correction was conducted to analyse the differences between the mean totals of the questionnaires.

**Results:**

In this study, 126 occupational therapy students participated (response rate = 57.3%). The students reacted positively to EBP, although few were practicing EBP. The students believed that EBP resulted in the best clinical care for patients, but they lacked confidence in their own ability to apply EBP. The students in Cohort 5, who received extra instruction and assignments via the EBP work file, rated their EBP behaviour statistically lower than the students in Cohort 1, who did not receive extra training on the EBP work file.

**Conclusions:**

Additional EBP work file assignments were insufficient in terms of supporting students in the implementation of EBP during clinical placements. It is, therefore, important to facilitate the learning strategies of EBP skills and demonstrate how students can practise this competency during clinical placements. Including clinical instructors in EBP teaching and learning seems essential.

## Background

On an international scale, evidence-based practice is expected to be included in occupational therapy education [1]. Evidence-based occupational therapy is defined as “the client-centred enablement of occupation, based on client information and a critical review of relevant research, expert consensus and experience” [2]. Accordingly, evidence-based practice (EBP) includes information from the client, research and clinical experience. To implement EBP, practitioners are advised to follow an EBP process. The Sicily Statement on Evidence-based Practice [3] considers the EBP process as consisting of five steps: 1) identifying information needs and formulating answerable questions; 2) searching for relevant articles to answer questions; 3) critically appraising research articles; 4) applying the results to clinical practice and 5) evaluating performance. At Step four, the research evidence must be integrated with clients’ preferences and students clinical experiences to make clinical decisions [4].

Healthcare students report various barriers to EBP during clinical placements, which include lack of time, high workload, lack of support to carry out evidence-based work during clinical placements, students and clinical instructors prioritizing hands-on practice during clinical placements, lack of role models practicing evidence-based work in clinical placements and insufficient autonomy to change practices [5–9]. Stronge and Cahill [9] examined the knowledge, attitudes and behaviours of occupational therapy students towards EBP. Their results revealed that the students had a clear understanding of EBP and a willingness to practise it in the future, however, they had difficulty finding research evidence relevant for clinical practice. Crabtree, Justiss and Swinehart [10] examined the EBP knowledge and skills of master level students of occupational therapy before and after clinical placements. The results showed that the students’ knowledge and skills in EBP increased before their placement but declined after their clinical placement. This outcome indicates that master level students improved their EBP skills and knowledge after learning the process of EBP, but the students did not learn any strategies enabling them to retain and use these skills beyond the classroom [10]. Measures that may support healthcare students, including bachelor students of occupational therapy, in applying their knowledge of EBP to clinical placements are collaboration with peers and teachers with a focus on EBP [11], being taught the fundamental skills of EBP [3, 4] and clinical instructors practicing EBP and expecting the same of their students [5, 9, 11].

Systematic reviews report that EBP teaching should be multifaceted and clinically integrated, including assessment of the EBP process [12–14]. There are different approaches to teaching and learning EBP, such as standalone or single teaching courses, including workshops, conferences, lectures, journal clubs and e-learning. In addition, EBP should be integrated into theoretical and clinical courses [6, 13]. Teaching EBP during clinical placements may improve EBP skills, beliefs and attitude [14]. Questions remain about how to teach EBP throughout the occupational therapy curriculum [6, 13].

The EBP work file is a Microsoft Word document that guides the student through the five EBP stages [15, 16]. The EBP work file contains specific questions for each of the five steps such as 1) “Describe the clinical situation and your information requirements”, “Complete all relevant PICO elements”, 2) “Describe your search words and a combination of these”, 3) “Critical appraise the research evidence”, 4) “How can you integrate knowledge from research with clinical experiences and with patients’ values and preferences?” and 5) “If you have changed practice, describe and evaluate the changes”.

The authors of this study have observed that students struggle to apply EBP during clinical placements. To support second year occupational therapy students applying EBP, we introduced the EBP work file as mandatory written course work during clinical placements.

The aim of this study was to examine occupational therapy students’ attitudes and behaviours towards applying evidence-based practice during their second-year clinical placement. We compared cohorts who received training in EBP work files with those who did not receive such training.

## Methods

This study used a descriptive, cross-sectional comparative design [17].

### Data collection

Bachelor students were recruited from two occupational therapy programmes in Norway, located at the Western University of Applied Sciences, Bergen (HVL) and Oslo Metropolitan University (OsloMet). Data collection was carried out between 2015 and 2017. Five cohorts of second-year occupational therapy students were asked to answer two questionnaires, the EBP Beliefs Scale and the EBP Implementation Scale following their clinical placements. All students (*n* = 220) were invited to participate in the study, however, 126 students in total answered the questionnaires (Table 1). The authors asked participants to provide demographic data relating to their age, sex and previous education.

**Table 1:** Overview of students in each cohort following clinical placement (HVL and OsloMet)

Cohort	1	2	3	4	5
**Year of Data Collection**	2015	2016	2017	2015	2016
**Total No. of Students**	35	27	36	64	59
**Study Site**	HVL	HVL	HVL	OsloMet	OsloMet
**No. of Students answering questionnaires (response rate)**	30 (80.0%)	19 (70.4%)	23 (63.9%)	11 (17.5%)	43 (72.9%)
**Sex**					
Female	28	17	17	10	35
Male	2	2	6	1	8
**Years**					
20–30	29	19	21	10	37
31–40	1		2		6
41–50				1	
**Previous education**					
Bachelor’s degree	4		2	2	7
Health-related studies	3	1	1	2	2
**Given instruction on the EBP work file**	No	Yes	Yes	No	Yes
**Mandatory submission of the EBP work file**	No	No	Yes	No	Yes

All cohorts in the study received varying amounts of EBP teaching during their education. The teaching was mainly related to the first steps of the EBP process in terms of identification of information needs and formulation of answerable questions, searching for relevant articles to answer questions and critical appraisal of research articles. The majority of EBP teaching methods found in research, only include the first steps of the EBP process [4]. The application of the results in clinical practice and the evaluation of performance are less emphasized and are not regarded as a requirement during occupational therapy education both at HVL and OsloMet. To support the students in the application of all the steps of the EBP process during their clinical practice, we introduced the EBP work file [15, 16]. The EBP work file is a Word document, developed to support the students in conducting evidence-based work [16]. The students followed and completed every step of the EBP work process, using the EBP work file to support the implementation of EBP in their clinical practice. Students had the opportunity to ask for guidance in completing the EBP work file during clinical placements. Cohorts 1 and 4 did not receive any instruction on the EBP work file, however, Cohorts 2, 3 and 5 received standardized training for 3 h on how to use the EBP work file during clinical placement. The submission of the EBP work file for assessment was voluntary in the case of Cohort 2, while it was mandatory for Cohorts 3 and 5.

### Questionnaires – EBP Beliefs Scale and EBP Implementation Scale

To examine students’ attitudes and behaviours in relation to EBP during clinical placements, the students answered two standardized questionnaires, the EBP Beliefs Scale and the EBP Implementation Scale in paper format [18]. The questionnaires (originally in English) were described as having criteria-related and conceptual validity, and had been found to be reliable in several studies [18, 19]. They were translated into the Norwegian language in accordance with the World Health Organization’s (WHO) process of translation and adaptation of instruments [19]. Since the two scales had been translated into the Norwegian language and used in two previous research articles [19, 20], it was relevant to use them in this study.

The EBP Beliefs Scale consists of 16 questions related to self-reported attitudes of EBP. Examples of questions are: “I believe that EBP results in the best clinical care for patients”, “I believe that I can overcome barriers to implementing EBP” and “I am confident about my ability to implement EBP where I work”. The response categories range from 1 (strongly disagree) to 5 (strongly agree). The total score for the questionnaire ranges from 16 to 80. Higher scores indicate positive attitudes towards EBP [18, 19].

**Fig. 1:**
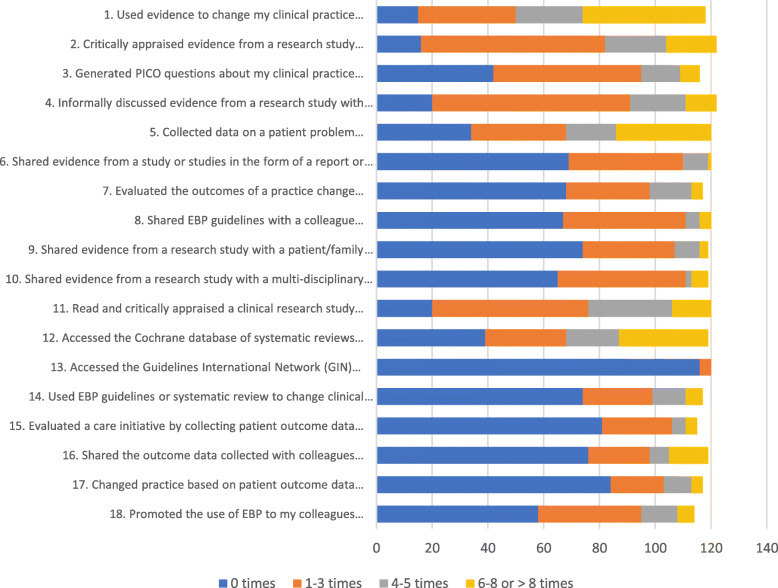
Students’ activities in terms of EBP behaviours which are requested in the EBP Implementation Scale

The EBP Implementation Scale comprises 18 statements measuring to what extent activities related to evidence-based behaviour are performed. Examples of questions are: “Promoted the use of EBP to my colleagues …” , “Used EBP guidelines or systematic review to change clinical practice where I work …” , and “Used evidence to change my clinical practice …” . All the questions relating to this tool are reported in Fig. 1. The scale’s response categories are distributed across a five point scale where zero is “zero times”, one is “1–3 times”, two is “4–5 times”, three is “6–8 times” and four is “> eight times”. The total score for the questionnaire ranges from 0 to 72 and higher scores reflect a more positive attitude in relation to EBP [18, 19].

### Statistical analysis

The analyses applied included descriptive statistics and calculation of the frequencies, percentages, means and standard deviations of the participating students’ scores across the EBP Beliefs and EBP Implementation Scales. Statistical analysis was conducted using one-way analysis of variance (ANOVA) with Bonferroni correction, to analyse differences in the mean totals for the EBP Beliefs and EBP Implementation Scales across all cohorts. For all analyses, the significance level was set at 5%, and statistical analysis was completed using the Statistical Package for Social Science (SPSS) data program, version 26 [21]. Two questions on the EBP Beliefs Scale were negatively formulated and the values of these two questions were reversed ahead of the analysis.

### Ethics

The students were informed about the purpose of this study, and that all information was anonymized. By answering the questionnaires, the students gave their consent to participate in the study. The authors did not collect sensitive information or data that could be linked to individual persons, therefore, ethical approval was not required under Norwegian regulations [22].

## Results

In all, 126 of 220 students participated (57.3% response rate). The response rate varied between the different cohorts, from 85.0 to 17.5% (Table 1). Those who completed the post-placement questionnaires included 107 females and 19 males. The age of the participants was reported in age groups, not in actual years of age; 116 students were between 20 and 30 years, nine students between 31 and 40 year and one student between 41 and 50 year. Fifteen students already had a bachelor’s degree and nine reported former involvement in health-related studies.

On the EBP Beliefs Scale, the mean score was 56.7 (standard deviation (SD) = 7.6, range = 39–74). The percentage of students who disagreed or strongly disagreed and agreed or strongly agreed with the questions is in Table 2. In all, 92.9% agreed that EBP results in the best clinical care for patients and 89.7% of the students believed that critical appraisal is an important step in the EBP process. Only 15.1% reported being sure about how to measure the outcome of clinical care, while 24.4% of the students disagreed that EBP is too time consuming (reverse scored). A comparison of the five cohorts using ANOVA with Bonferroni correction indicated no statistical difference between Cohorts 1 and 4 (who did not receive training on the EBP work file) and Cohorts 2, 3 and 5 (who did receive training in use of the EBP work file during clinical placements).

**Table 2:** Percentages of EBP Beliefs Scale scores of second-year occupational therapy students

EBP Beliefs Scale Questions	Total n (%)	Disagree or strongly disagree n (%)	Agree or strongly agree n (%)
1. I believe that EBP results in the best clinical care for patients.	123 (97.6)	2 (1.6)	117 (92.9)
2. I am clear about the steps of EBP.	123 (97.6)	9 (7.3)	94 (74.6)
3. I am sure that I can implement EBP.	123 (97.6)	12 (9.8)	79 (62.7)
4. I believe that critically appraising evidence is an important step in the EBP process.	120 (95.2)	1 (0.8)	113 (89.7)
5. I am sure that evidence-based guidelines can improve clinical care.	122 (96.8)	1 (0.8)	103 (81.7)
6. I believe that I can search for the best evidence to answer clinical questions in a time-efficient way.	122 (96.8)	17 (13.9)	47 (37.3)
7. I believe that I can overcome barriers to implementing EBP.	117 (92.9)	1 (0.8)	73 (57.9)
8. I am sure that I can implement EBP in a time-efficient way.	121 (96.0)	27 (22.3)	34 (27.0)
9. I am sure that implementing EBP will improve the care that I deliver to my patients.	120 (95.2)	3 (2.5)	100 (79.4)
10. I am sure about how to measure the outcomes of clinical care.	119 (94.4)	34 (28.6)	19 (15.1)
11. I believe that EBP takes too much time.	120 (95.2)	42 (33.4)	31 (24.6)
12. I am sure that I can access the best resources in order to implement EBP.	122 (96.8)	14 (11.5)	36 (28.5)
13. I believe that EBP is difficult.	122 (96.8)	36 (28.5)	49 (38.8)
14. I know how to implement EBP sufficiently to make practice changes.	120 (95.2)	27 (22.3)	93 (73.8)
15. I am confident about my ability to implement EBP where I work.	122 (96.8)	36 (29.5)	39 (31.0)
16. I believe that the care that I deliver is evidence based.	120 (95.2)	39 (32.5)	81 (64.2)

In terms of EBP Implementation, the mean total score was 15.4 (SD = 9.0, range = 1–51), which is considered low. Figure 1 gives an overview of the students reporting on each question of the EBP implementation questionnaire. The questions which recorded the highest frequency of carrying out a task (4–5 or 6–8 or > 8 times) were Questions 1 (57.6%), 5 (43.3%) and 12 (42.9%). The students reported using evidence to change practice, collected data on patient problems and accessed the Cochrane Library. The response ‘zero times’ was highest in relation to Question 13 (96.7%), 15 (70.4%) and 17 (71.8%). This indicates that accessing Guidelines International Network (GIN) and evaluating and changing practice on patient outcome data, is most difficult for students.

A comparison of the five cohorts indicated that students in Cohort 5 (who received training in using EBP work files) had statistically lower scores on the EBP Implementation Scale than students from Cohort 1 (who did not have extra instruction) (Table 3). There was no significant statistical difference between the other cohorts.

**Table 3:** Results of the ANOVA Bonferroni test, comparing the answers of the five cohorts of bachelor students from the EBP Implementation Scale

Cohorts: HVL or OsloMet	Cohorts: HVL or OsloMet	Mean Difference	Std. Error	Sig.	95% Confidence Interval	
					Lower Bound	Upper Bound
Cohort 1 HVL	Cohort 2	4.9	2.9	0.9	−3.4	13.2
	Cohort 3	6.7	2.7	0.1	−0.9	14.4
	Cohort 5	7.3	2.3	0.0^a^	0.6	14.1
Cohort 2 HVL	Cohort 1	−4.9	2.9	0.9	−13.2	3.4
	Cohort 4	1.4	4.5	1.0	−11.5	14.3
Cohort 3 HVL	Cohort 1	−6.7	2.7	0.1	−14.4	0.9
	Cohort 4	−0.5	4.4	1.0	−13.0	12.1
Cohort 4 OsloMet	Cohort 2	−1.4	4.5	1.0	−14.3	11.5
	Cohort 3	0.5	4.4	1.0	−12.1	13.0
	Cohort 5	1.1	4.2	1.0	−11.0	13.0
Cohort 5 OsloMet	Cohort 1	−7.3	2.3	0.0^a^	−14.1	−0.6
	Cohort 4	−1.1	4.2	1.0	−13.0	10.9

^a^The mean difference is significant at the 0.05 level

## Discussion

Evidence-based practice should be included in occupational therapy curricula [1, 12], to support students’ utilization of EBP in their clinical practice. The results indicated that the occupational therapy students regarded EBP as important for clinical practice, but they experienced problems in applying EBP. Providing students with training in using the EBP work file was not enough to strengthen their self-perceived ability to apply EBP to clinical practice.

The average scores in other comparable studies of the EBP Belief Scale vary from 53.0 to 64.1 (maximum 80) [19], which is similar to our results. Our results indicate that bachelor level students of occupational therapy believe that EBP results in the best clinical care for patients, although they thought EBP was too time consuming and perceived it as difficult to apply. Similar barriers have been found in previous research [5–9]. The EBP work file is designed to help and assist students in every step of the EBP process [16], although it does not facilitate student’s application of EBP in clinical situations. To support the student’s application of EBP in clinical placement, the students probably need a more concrete and relevant EBP assignment related to real world problems. This aligns with previous research which emphasizes that EBP teaching should be meaningful and of relevance to the students [1].

EBP teaching for occupational therapy students at OsloMet and HVL seems to concentrate on the first steps of EBP, e.g. searching for, finding and critically appraising evidence, with less emphasis on integrating and using research evidence in conjunction with clinical and user experiences. The use and translation of research evidence into clinical practice needs to be prioritized [11, 13], including enabling students to apply all the steps of the EBP process [3]. More emphasis on the last step of the EBP process, namely integrating and using research evidence, could boost student’s motivation, enabling them to understand and acknowledge that EBP makes a difference in relation to best practice.

Previous research indicates that students have trouble retaining and using EBP skills beyond the classroom [9, 10]. Thus, the students may have perceived the EBP work file as a university assignment, unrelated to clinical practice. When assignments are regarded as separate from clinical practice, the translation of EBP is not assured [11]. Teaching strategies, such as using relevant, authentic cases which include the natural complexities of the world, seem important [11]. Crabtree et al. [10] suggested using practice scenarios facilitated by clinical instructors, to make the teaching more relevant and realistic for the students. These suggestions may contribute to closing the gap between theoretical and practical knowledge, although this may not guarantee that EBP is integrated into a student’s clinical work. An online tracking of the student’s EBP work could potentially enable faculty and clinical instructors to support students in the application of EBP during clinical placement.

EBP implementation was low in terms of the occupational therapy students, although our results are slightly higher than those reported in a study by Stokke et al. [20], in which Norwegian nurses recorded an average score of 7.8 (compared to 15.4 in the present study). The lack of utilization of EBP on the part of occupational therapists’ may also be found in a systematic review of occupational therapists’ implementation of EBP [23]. This indicates that the implementation of EBP may be challenging for healthcare professionals, including occupational therapists, and consequently, they have difficulty acting as EBP role models for students. Several studies have reported on the lack of support from clinical instructors, amongst others, as a barrier to EBP, affecting students in their clinical placements [6, 9, 10, 24]. Considering that the implementation process, in general, is demanding for healthcare professionals, it is, therefore, not surprising that occupational therapy students have difficulty in applying EBP. Supporting clinical instructors to be confident in terms of their own competence when teaching EBP and to present themselves as role models for students, can assist students in applying EBP during clinical placements [1].

The translation of evidence and implementation of EBP in clinical placements is influenced by several factors, such as the access to research evidence, deciding on the applicability of the evidence and the need for habits to change [12]. The accessibility of research evidence has been highlighted as a significant barrier for occupational therapists’ [23]. Students have easy access to a variety of research evidence via the university library, which could benefit the clinical instructors. Having access to the research evidence, the students and clinical instructors could read and discuss this evidence and decide whether to include the results from research in practice, e.g., conducting a journal club. Journal clubs may serve to support the implementation of EBP [11, 12, 23].

Teaching and learning EBP form just one element of the training of occupational therapy students during clinical placements. Competing demands and other priorities have been found to act as a barrier to applying EBP during clinical placements [5]. In this study, the EBP work file, provided as an assignment was insufficient to enable occupational therapy students to increase the implementation of EBP during clinical placements. EBP assignments probably need to be introduced and worked on during clinical placements, with clinical instructors actively involved [5] in supporting the students, to translate the research evidence into practice [11].

### Implications for occupational therapy education

We argue that including an EBP-based assignment during clinical placements is insufficient in terms of boosting students’ confidence in engaging with EBP. EBP teaching and its associated assignments should include the active involvement of both students and clinical instructors. Had the EBP work file been better integrated as part of the clinical placement and been known to the clinical instructors, the outcome in relation to the students’ self-perceived attitudes and behaviours towards applying EBP to clinical placements, may have been better. Previous research has noted the importance of including clinical instructors in the planning and delivery of EBP curricula activities during clinical placements [10]. For example, the faculty should involve and collaborate with clinical instructors with a view to giving students opportunities to present and discuss the results of research articles and to implement EBP in clinical placements. One recommendation to overcoming EBP barriers, is to include a structural incorporation of EBP in clinical placements [1]. Involving clinical instructors in the inclusion of EBP in clinical placements, may contribute to closing the gap between theoretical teaching and practice, enabling students to translate research evidence into practice and potentially effect practice change [11]. Clinical placement provides a unique opportunity of learning the implementation of EBP in patient treatment and can promote motivation for lifelong learning.

### Study strengths and limitations

Participants in this study included bachelor students from two different occupational therapy programmes in Norway from three different year groups, providing a wider cross-section of students’ self-reported attitudes and behaviour towards EBP. However, the fact that only two study sites in one country were used, render the sample small, therefore, the results should be treated with caution. The study’s limitations include a limited sample size and the lack of a control group. This may have led to differences in the results according to the confounding variables. However, the use of ANOVA with Bonferroni correction has controlled for confounding variables.

The authors have considered whether the questionnaires may have been difficult to answer due to the role of students in clinical placements, especially questions related to implementation of EBP, for example, “[I have] [u] sed evidence to change my clinical practice”. A barrier to employing an evidence-based method is insufficient autonomy to be able to change practice [8]. Bachelor level students of occupational therapy would not be expected to change the practice of the institution while on placement, but they could be engaged in the process of change in consultation with their clinical instructor. Therefore, the question could potentially be changed to “I have used evidence and changed practice together with my clinical instructor”, which might be more appropriate for students.

The translation of both questionnaires into the Norwegian language was carried out in accordance with the WHO’s process of translation and adaptation of instruments [19]. However, the questionnaires have not been tested for cross-cultural validity, which may be a potential limitation in this study. Future research could use control groups and interviews with both students and clinical instructors to investigate further how best to implement EBP teaching strategies during clinical placements. An online tracking of the EBP work file could also reinforce the results of the study.

## Conclusion

This study’s findings indicate a positive attitude towards EBP on the part of bachelor level students of occupational therapy, however, they found it difficult to practise and implement EBP during clinical placements. Simply giving students an EBP-based assignment using the EBP work file does not enable them to implement EBP while on placement. This fact underlines the importance of teaching students EBP skills, which would enable them to use EBP when working with patients. It is important and necessary to include clinical instructors in the planning and teaching of EBP during clinical placements. Hence, cooperation between faculty and clinical instructors on curricula development must be given high priority in order to increase confidence in EBP and a positive attitude in relation to EBP, on the part of the students.

## Data Availability

The dataset used and/or analysed during this study is available from the corresponding author upon reasonable request.

## Abbreviations

ANOVA: One-way analysis of variance
EBP: Evidence-based practice
HVL: Western Norway University of Applied Sciences
OsloMet: Oslo Metropolitan University
SPSS: Statistical Package for Social Science
WHO: World Health Organization
